# Supplementary material for: Months-long tracking of neuronal ensembles spanning multiple brain areas with Ultra-Flexible Tentacle Electrodes
Source: Nat Commun. 2024 Jun 6;15:4822. doi: 10.1038/s41467-024-49226-9 (PMC11156863; doi:10.1038/s41467-024-49226-9)
Supplement: Supplementary file 1 — Supplementary Material [file 41467_2024_49226_MOESM1_ESM.pdf]

Months-long tracking of neuronal ensembles spanning multiple brain areas with Ultra-Flexible Tentacle Electrodes

Tansel Baran Yasar[1,2,5], Peter Gombkoto[1,2,5], Alexei L. Vyssotski[1,2], Angeliki D. Vavladeli[1,2], Christopher M. Lewis[2,3], Bifeng Wu[1,2], Linus Meienberg[1], Valter Lundegardh[1], Fritjof Helmchen[2,3,4], Wolfger von der Behrens[1,2], Mehmet Fatih Yanik[1,2,*]

[1]Institute of Neuroinformatics, ETH Zurich & University of Zurich, Zurich, Switzerland. [2]Neuroscience Center Zurich, University of Zurich & ETH Zurich, Zurich, Switzerland. [3]Brain Research Institute, University of Zurich, Zurich, Switzerland. [4]University Research Priority Program (URPP), Adaptive Brain Circuits in Development and Learning, University of Zurich, Zurich, Switzerland. [5]These authors contributed equally: Tansel Baran Yasar, Peter Gombkoto. [*]Correspondence: yanik@ethz.ch

Supplementary Material

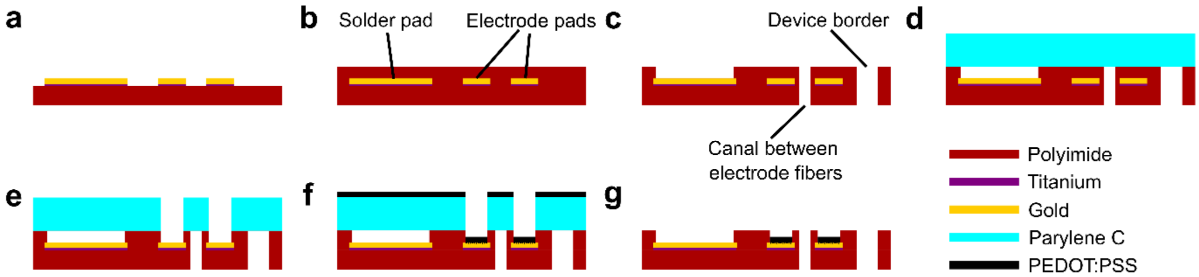

Supplementary Figure 1: Electrode fabrication layers. **A:** Titanium/gold is patterned on the base polyimide layer to define the electrode contacts, solder pads, and the connecting wires between them. **B:** An insulating polyimide layer is coated on metal features. **C:** Polyimide between the electrode fibers, on the device borders, and above the solder pads is etched away. **D:** The wafer is coated with a sacrificial layer of parylene C. **E:** The sacrificial parylene layer and the insulating polyimide layer above the electrode contacts are etched away. **F:** The gold on the electrode contacts is roughened by a gold etchant, and the wafer is spin-coated with PEDOT:PSS. **G:** The sacrificial parylene C layer is peeled off to eliminate the excess PEDOT:PSS.

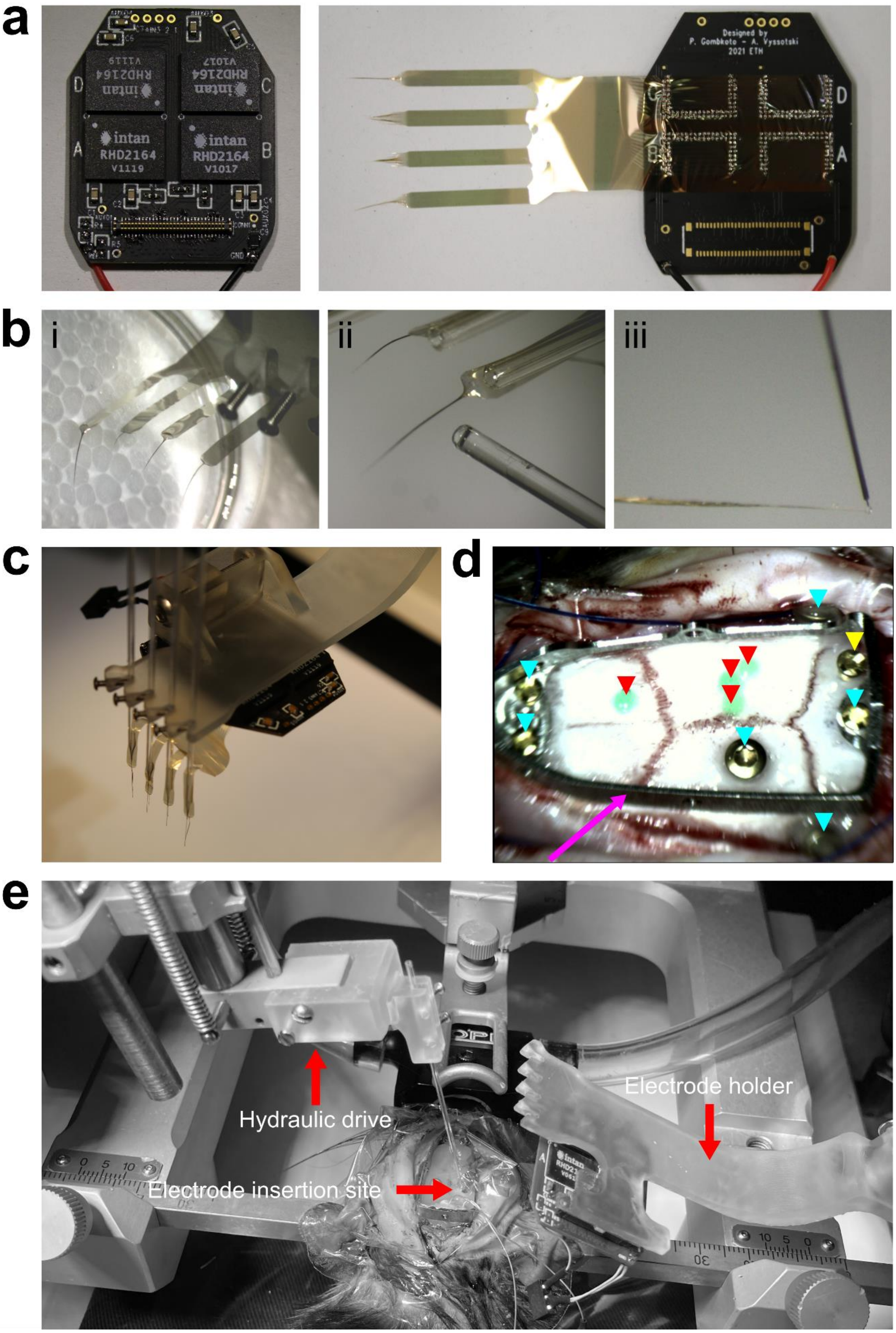

Supplementary Figure 2: Details of the electrode array assembly and implantation. **A:** The custom 256-channel Intan-based headstage used for the chronic recordings. Left: Front side

of the board with the four Intan RHD2164 chips. Right: The ultra-flexible electrode array is soldered to the backside. **B:** Steps of the electrode assembly. i: The electrode bundles are inserted into the PEG solution as a stiffener before the silk coating. ii: The electrode bundles are coated with silk fibroin solution. iii: The loop at the end of the electrode bundle is approached with a tungsten wire using a stereotaxic frame. **C:** The picture of the fully-assembled 4-bundle 256-channel electrode array. **D:** The surface of the rat skull with the titanium base (magenta arrow), titanium screws (cyan triangles), reference screw (yellow triangle), and the craniotomies for the electrode insertion are covered with silicon elastomer (red triangles). **E:** Implantation of a UFTE bundle into the brain.

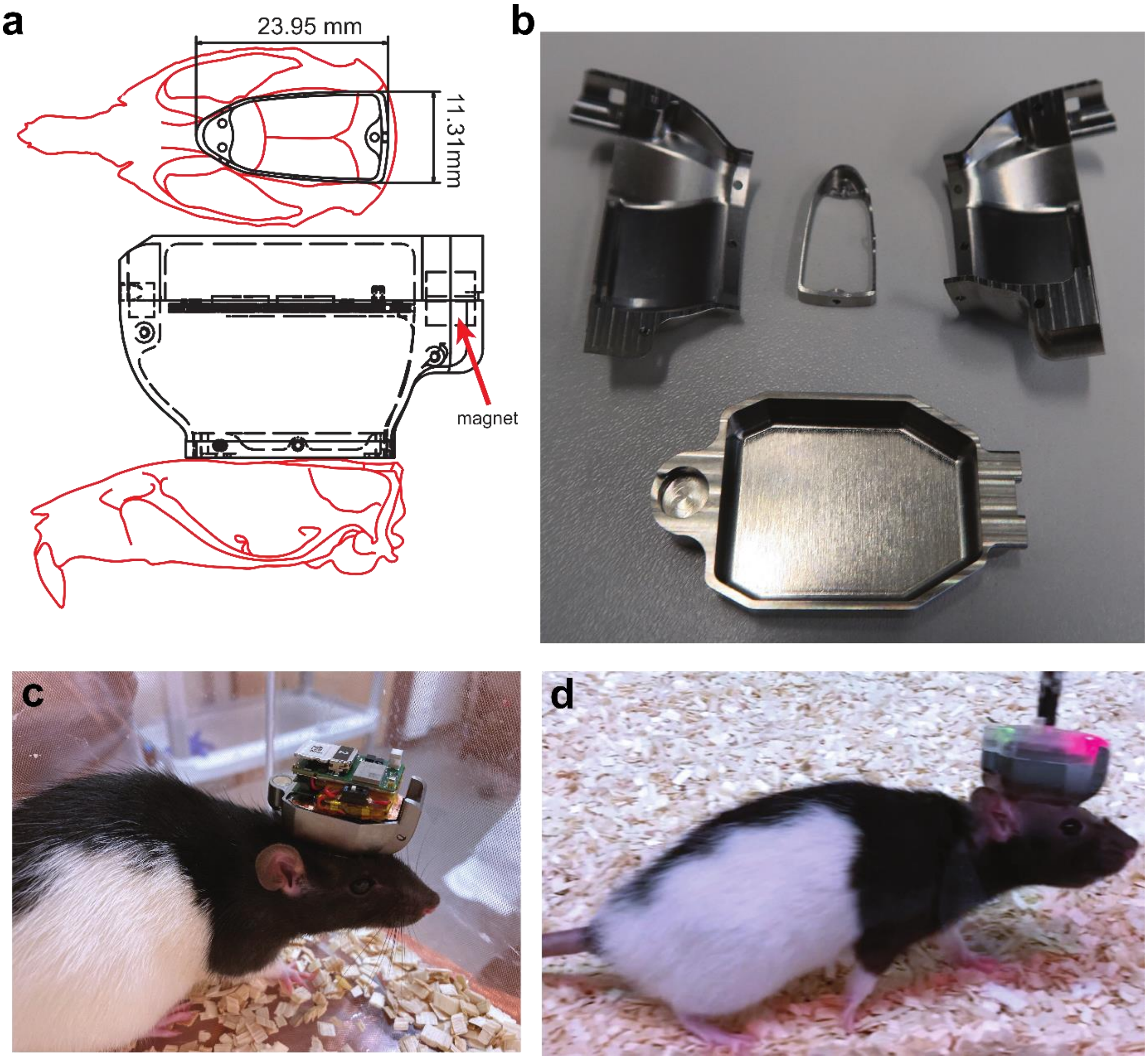

Supplementary Figure 3: Ultralight (13 g) TitaniumHelmet. **A:** Top: The base design on a rat's skull. Bottom: schematic wireframe side view with hidden edges of the cap and head stage on a rat’s skull. Red arrow indicates the place of the caudal magnet. **B:** Machined titanium parts. Left shell, base, right shell, and top enclosure of the cap. **C:** Rat with wireless 512-channel electrophysiology logger and battery, which can save data from the head stage to SD card for up to one hour. **D:** A wired version of the recording involves a transparent 3D-printed top part connected to the TitaniumHelmet. This part establishes a wired connection to the head stage, incorporating rostral (red) and caudal (green) LEDs for tracking animal orientation during recording.

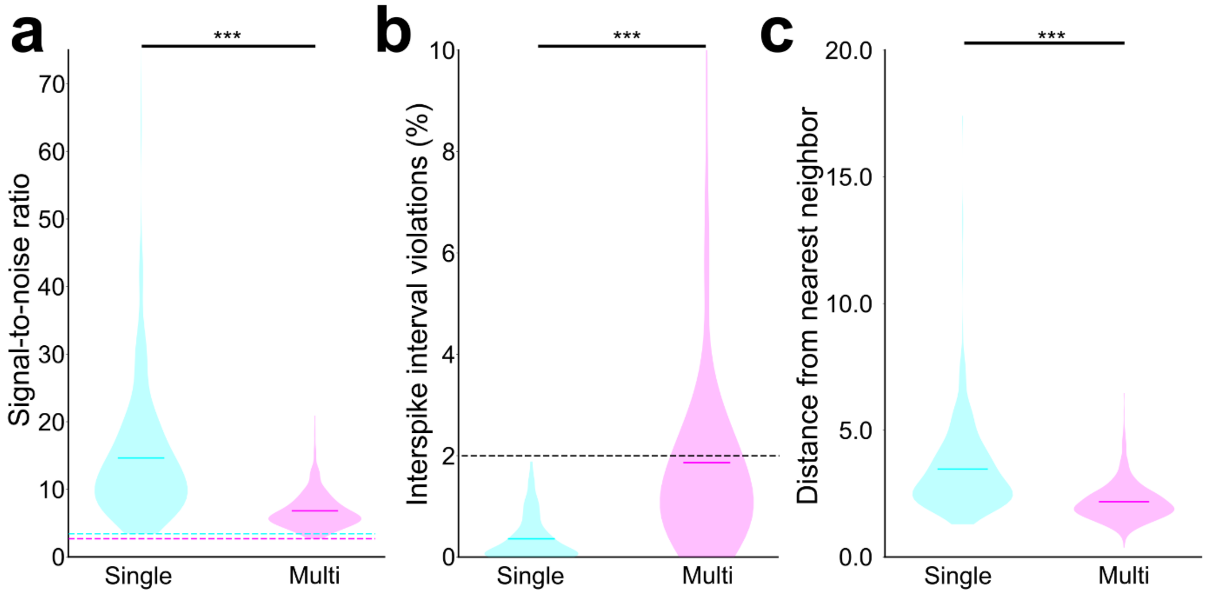

Supplementary Figure 4: The quality scores distinguishing between single and multi-units. **A:** Signal-to-noise ratio (SNR) distributions of single vs multi-units. The dashed magenta line indicates the SNR threshold for detecting spikes, whereas the dashed cyan line indicates the SNR threshold for accepting single units. $p=1.26\times10^{-64}$, two-sided Wilcoxon's Rank-Sum test. **B:** Distributions of the percentage of interspike intervals violating a 2 ms minimum for single- vs. multi-units. The dashed line indicates the 2% limit of interspike interval violations allowed for single units, above which the single units are rejected. $p=6.55\times10^{-67}$, two-sided Wilcoxon's Rank-Sum test **C:** Distributions of Mahalanobis distances from the nearest neighbors for single vs. multi-units. $p=2.46\times10^{-49}$, two-sided Wilcoxon's Rank-Sum test. In all panels, *** indicates $p < 0.001$ for the null hypothesis that the compared distributions are identical. Number of single units is n=445. Number of multi units is n=345. Source data are provided in the Supplementary Data file.

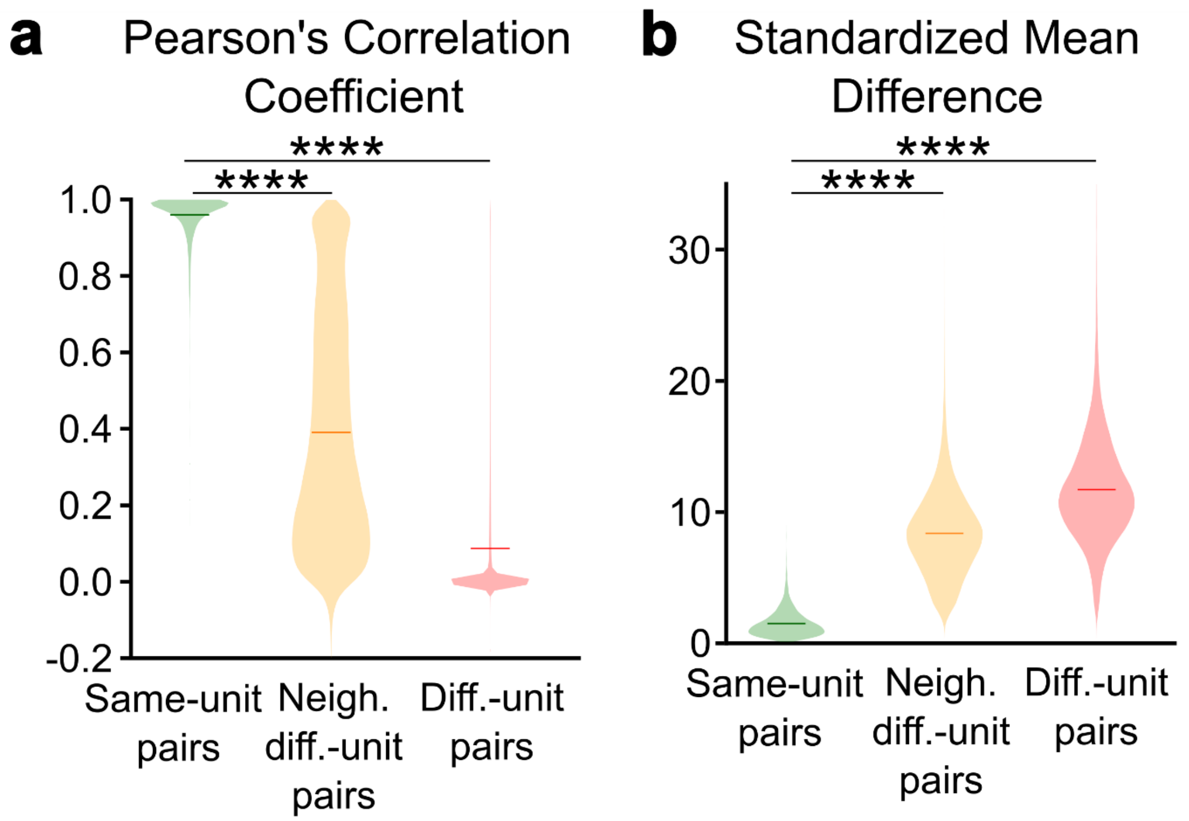

Supplementary Figure 5: The metrics used for verifying single-unit stability across sessions. **A:** Distributions of Pearson's correlation coefficients across "same-unit pairs" (green), "neighboring different-unit pairs" (yellow), and "different-unit pairs" (red). **B:** Distributions of the standardized mean differences across "same-unit pairs" (green), "neighboring different-unit pairs" (yellow), and "different-unit pairs" (red). n=5225 for "same-unit pairs", n=741754 for "neighboring different-unit pairs", n=3501090 for "different-unit pairs". In both panels, solid lines in the violin plots indicate the mean values, and **** shows $p < 0.0001$ for the null hypothesis that the compared distributions are identical ($p < 10^{-100}$ with two-sided Wilcoxon Rank-Sum Test). Source data are provided in the Supplementary Data file.

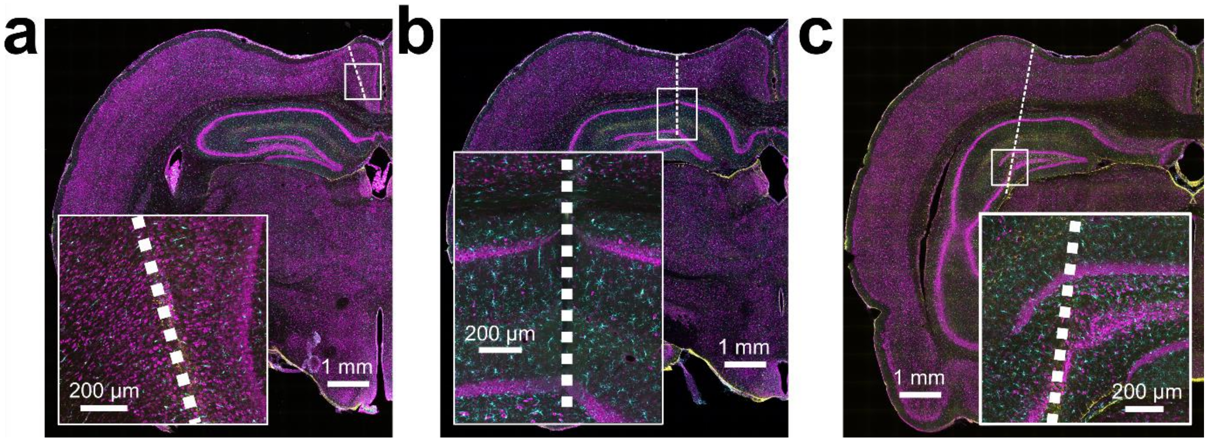

Supplementary Figure 6: Histological staining of brain slices from different implant sites 3.5 months post-implantation, where **A:** Implant targeted at the retrosplenial cortex, **B:** Implant targeted at dorsal hippocampus, **C:** Implant targeted at intermediate hippocampus. In all cases, the inset shows a magnified view of the box. Dashed lines indicate the space that was formerly occupied by the electrode bundle. Magenta shows neurons stained by fluorescent Nissl (Neurotrace 640/660), cyan shows microglia stained by anti-IBA-1, and yellow shows activated astrocytes stained by anti-GFAP.

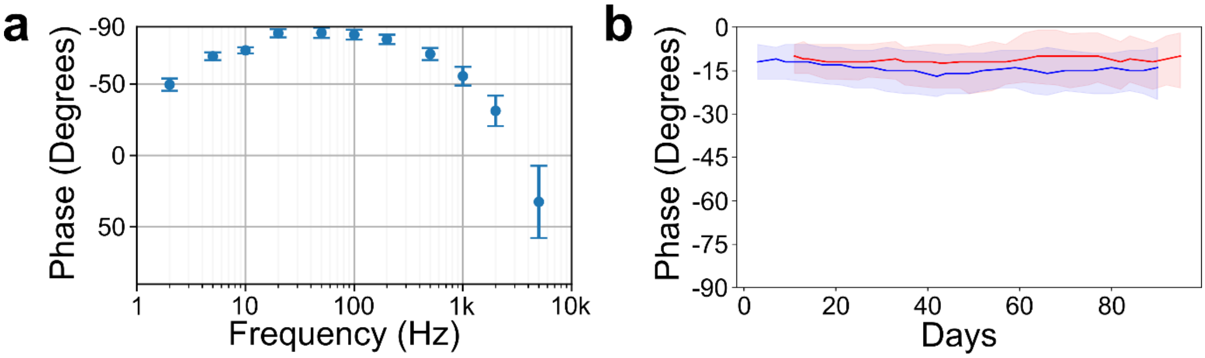

Supplementary Figure 7: Characterization of the impedance phase from the recording contacts of the UFTEs. **A:** Impedance phase spectroscopy of the 256-channel UFTE in Ringer's solution pre-assembly (mean ± s.d., n=243 recording contacts)**.** Outliers are excluded in the same way as in Fig. 2c. **B:** Electrode-tissue impedance phases during 3.5 months in two rats at 1 kHz frequency (red: Rat #1, blue: Rat #2)**.** For each data trace, line shows the median and filled area shows the interquartile range of impedance phases across functional recording contacts (n=184,181,185 contacts on days 27, 61, 95 for Rat #1 and n=236, 234, 230 contacts on days 24, 59, 90 for Rat #2). Source data are provided in the Supplementary Data file.

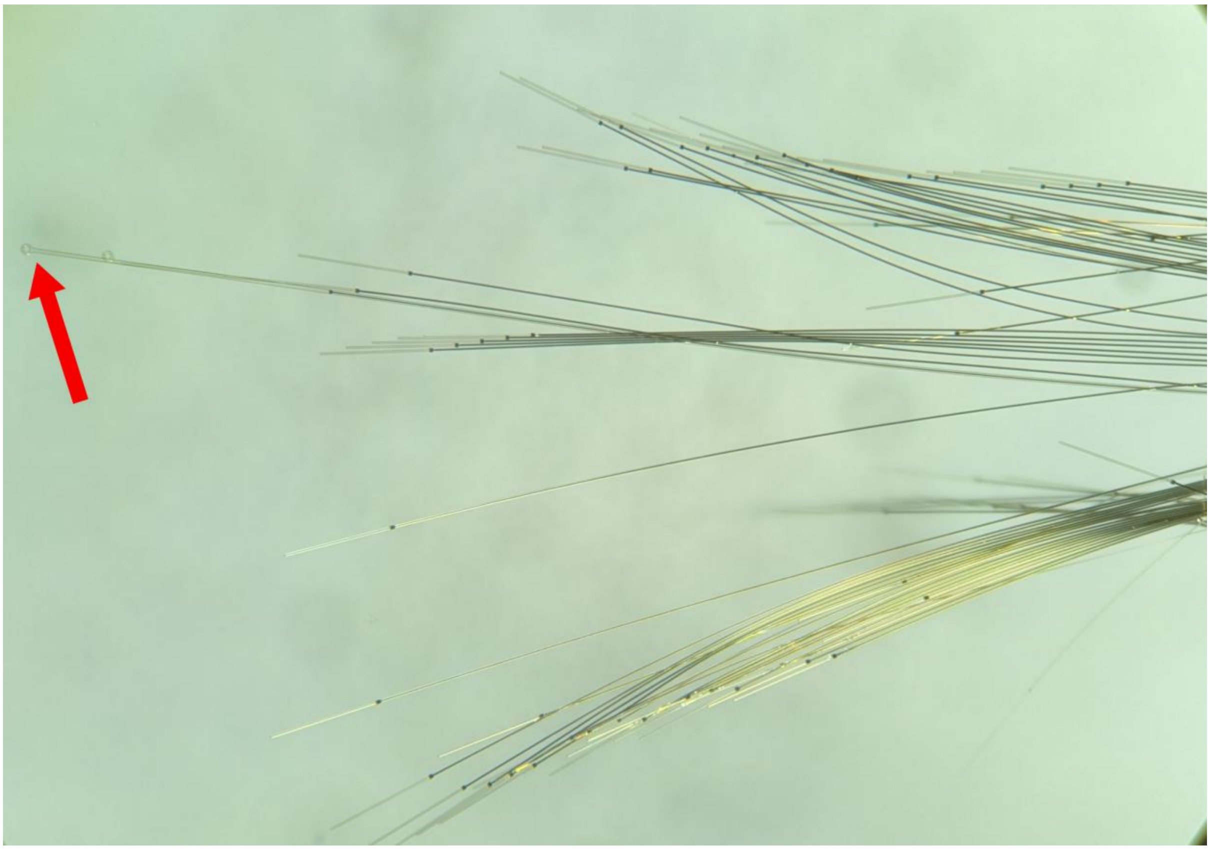

Supplementary Figure 8: A larger version of Fig. 2a(v) showing the electrode fibers of a UFTE bundle floating in distilled water. The red arrow points to the primary loop at the tip of the bundle.

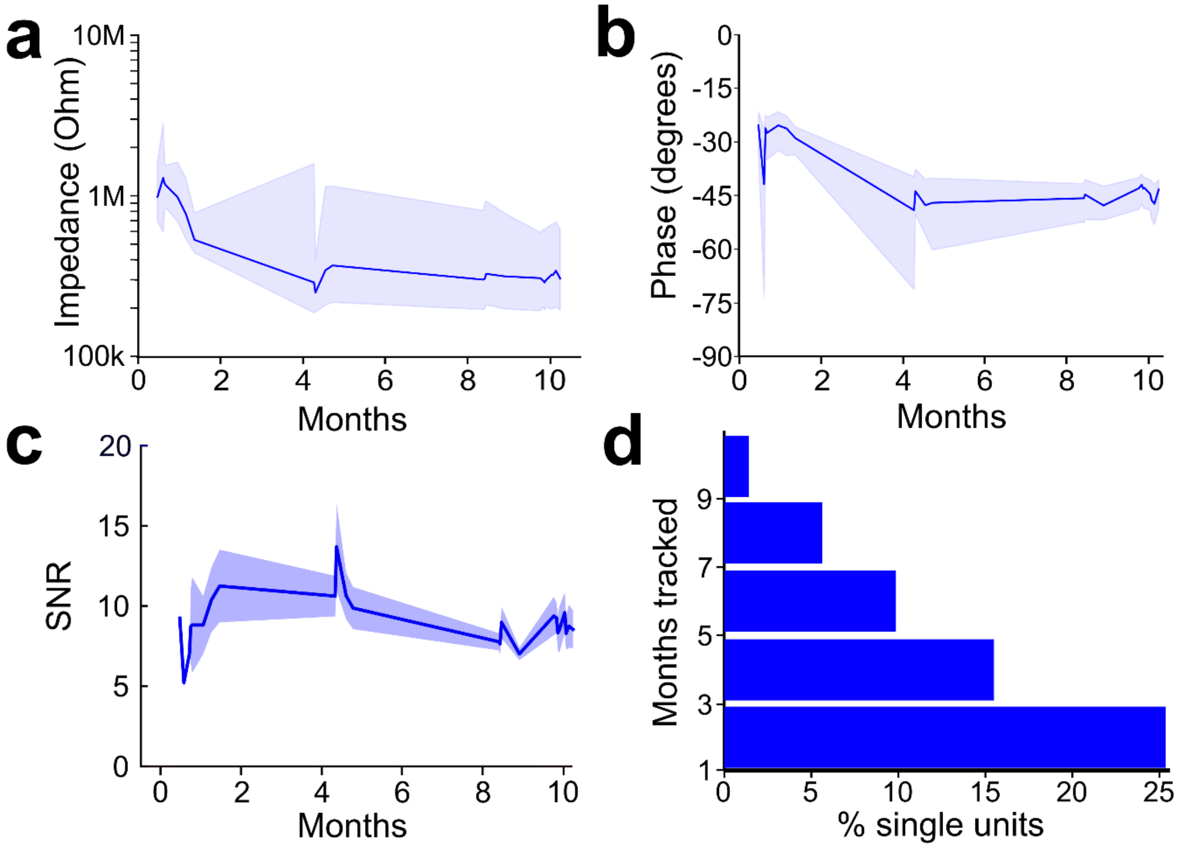

Supplementary Figure 9: Stability of the recordings from the mouse brain. **A:** Electrode-tissue impedance magnitudes during 10 months in the mouse brain at 1 kHz frequency**.** Line shows the median and filled area shows the interquartile range of impedances across functional recording contacts (n=61,63,62,63,63 recording contacts on post-implantation days 29,50,147,268,301). **B:** Electrode-tissue impedance phases during 10 months in the mouse brain at 1 kHz frequency**.** For each data trace, the line shows the median and the filled area shows the interquartile range of impedance phases across functional recording contacts (n=61,63,62,63,63 recording contacts on post-implantation days 29,50,147,268,301). **C:** Signal-to-noise ratio of single units recorded from the mouse brain across 10 months. Graph shows mean ± s.e.m. **D:** The longitudinal tracking durations of single units recorded from the mouse brain.

Supplementary Table 1: Comparison of different insertion types, depths, speeds into 0.9% agar we tested using comparable glue footprints.

**Shuttle diameter (μm)**	**Method for tethering the shuttle to the fiber bundle**	**Target depth (mm)**	**Insertion speed (μm/s)**	**Reached depth (mm)**	**Success rate**
50	Gelatin*	5	100	2-3	7/12
50	Silk fibroin	5	100	5	7/12
25	Gelatin+silk fibroin	5	100	5	11/12
25	Gelatin+silk fibroin	5	20	5	10/12
25	UFTE	5	100	5	6/6
25	UFTE	5	20	5	6/6

(Gelatin here refers to the gelatin/glycerol/PEG 400 coating developed by Agorelius et al[1].)

References

1. Agorelius, J. *et al.* An array of highly flexible electrodes with a tailored configuration locked by gelatin during implantation—initial evaluation in cortex cerebri of awake rats. *Front. Neurosci.* **9**, (2015).
