# Supplementary material for: Months-long tracking of neuronal ensembles spanning multiple brain areas with Ultra-Flexible Tentacle Electrodes
Source: Nat Commun. 2024 Jun 6;15:4822. doi: 10.1038/s41467-024-49226-9 (PMC11156863; doi:10.1038/s41467-024-49226-9)
Supplement: Supplementary file 3 — Description Of Additional Supplementary File [file 41467_2024_49226_MOESM3_ESM.pdf]

Description of Additional Supplementary File

Supplementary Video 1: Video of a freely moving rat during a recording session with local field potential/spike data simultaneously recorded from multiple brain areas.

Supplementary Video 2: Video of 3 group-housed rats implanted with ultra-light Titanium Helmets
