# Supplementary material for: Months-long tracking of neuronal ensembles spanning multiple brain areas with Ultra-Flexible Tentacle Electrodes
Source: Nat Commun. 2024 Jun 6;15:4822. doi: 10.1038/s41467-024-49226-9 (PMC11156863; doi:10.1038/s41467-024-49226-9)
Supplement: Supplementary file 6 — Reporting Summary [file 41467_2024_49226_MOESM6_ESM.pdf]

nature portfolio

Corresponding author(s): Mehmet Fatih Yanik

Last updated by author(s): May 23, 2024

Reporting Summary

Nature Portfolio wishes to improve the reproducibility of the work that we publish. This form provides structure for consistency and transparency in reporting. For further information on Nature Portfolio policies, see our Editorial Policies and the Editorial Policy Checklist.

Statistics

For all statistical analyses, confirm that the following items are present in the figure legend, table legend, main text, or Methods section.

n/a	Confirmed	
☐	☒	The exact sample size (*n*) for each experimental group/condition, given as a discrete number and unit of measurement
☐	☒	A statement on whether measurements were taken from distinct samples or whether the same sample was measured repeatedly
☐	☒	The statistical test(s) used AND whether they are one- or two-sided<br>*Only common tests should be described solely by name; describe more complex techniques in the Methods section.*
☒	☐	A description of all covariates tested
☒	☐	A description of any assumptions or corrections, such as tests of normality and adjustment for multiple comparisons
☐	☒	A full description of the statistical parameters including central tendency (e.g. means) or other basic estimates (e.g. regression coefficient) AND variation (e.g. standard deviation) or associated estimates of uncertainty (e.g. confidence intervals)
☐	☒	For null hypothesis testing, the test statistic (e.g. *F*, *t*, *r*) with confidence intervals, effect sizes, degrees of freedom and *P* value noted<br>*Give P values as exact values whenever suitable.*
☒	☐	For Bayesian analysis, information on the choice of priors and Markov chain Monte Carlo settings
☒	☐	For hierarchical and complex designs, identification of the appropriate level for tests and full reporting of outcomes
☐	☒	Estimates of effect sizes (e.g. Cohen's *d*, Pearson's *r*), indicating how they were calculated

Our web collection on statistics for biologists contains articles on many of the points above.

Software and code

Policy information about availability of computer code

Data collection	Intan RHX 3.1 (Intan Technologies), cellSens (Olympus LS)
Data analysis	Matlab 2023a (MathWorks), Python 3, ImageJ (NIH), cellSens (Olympus LS), JRCLUST 4.0.0,<br>Custom code provided in: https://github.com/Neurotechnology-at-ETH-Zurich/UFTE_paper

For manuscripts utilizing custom algorithms or software that are central to the research but not yet described in published literature, software must be made available to editors and reviewers. We strongly encourage code deposition in a community repository (e.g. GitHub). See the Nature Portfolio guidelines for submitting code & software for further information.

Data

Policy information about availability of data

All manuscripts must include a data availability statement. This statement should provide the following information, where applicable:

- Accession codes, unique identifiers, or web links for publicly available datasets
- A description of any restrictions on data availability
- For clinical datasets or third party data, please ensure that the statement adheres to our policy

All data supporting the findings of this study are available within the article and its supplementary files. Additional data is deposited to the Zenodo repository at: https://zenodo.org/records/11236154. Any additional requests for information can be directed to, and will be fulfilled by, the corresponding authors. Source data are provided with this paper.

Human research participants

Policy information about [studies involving human research participants and Sex and Gender in Research.](#)

Reporting on sex and gender	n/a
Population characteristics	n/a
Recruitment	n/a
Ethics oversight	n/a

Note that full information on the approval of the study protocol must also be provided in the manuscript.

Field-specific reporting

Please select the one below that is the best fit for your research. If you are not sure, read the appropriate sections before making your selection.

☒ Life sciences ☐ Behavioural & social sciences ☐ Ecological, evolutionary & environmental sciences

For a reference copy of the document with all sections, see [nature.com/documents/nr-reporting-summary-flat.pdf](#)

Life sciences study design

All studies must disclose on these points even when the disclosure is negative.

Sample size	Number of single units = 445, number of electrode insertions = 7 in two rats for the single-unit stability/quality characterization and the neuronal ensemble analysis. No sample-size calculation was performed. 7 electrode insertions in different brain areas were considered to be sufficiently representative of the quality of the recordings with UFTEs.
Data exclusions	No data excluded as outliers. Spike clusters that were not identified as single units during the spike sorting process and did not pass the ISI violation criteria were identified as multi units and excluded from the ensemble and single-unit stability/quality characterization, as clearly stated in the Methods.
Replication	13 electrode bundles were inserted in 4 rats to verify the reproducibility of our electrode implantation method.
Randomization	This was not relevant to our study due to the absence of multiple conditions/groups to compare.
Blinding	The spike sorting process and the contributing author in charge of the manual curation of the sorting were blinded to the potential neuronal ensemble memberships of the single units. The ensemble analysis was performed by a different contributing author than who performed the spike sorting process.

Reporting for specific materials, systems and methods

We require information from authors about some types of materials, experimental systems and methods used in many studies. Here, indicate whether each material, system or method listed is relevant to your study. If you are not sure if a list item applies to your research, read the appropriate section before selecting a response.

Materials & experimental systems

n/a	Involved in the study	
☐	☒	Antibodies
☒	☐	Eukaryotic cell lines
☒	☐	Palaeontology and archaeology
☐	☒	Animals and other organisms
☒	☐	Clinical data
☒	☐	Dual use research of concern

Methods

n/a	Involved in the study	
☒	☐	ChIP-seq
☒	☐	Flow cytometry
☒	☐	MRI-based neuroimaging

Antibodies

Antibodies used	Rabbit-anti-IBA1 - (019-19741, FUJIFILM Wako), goat-anti-GFAP (ab53554, Abcam), donkey-anti-goat Alexa Fluor 405 nm (ab175664,

Antibodies used	Abcam), goat-anti-rabbit Alexa Fluor Plus 488 nm (A32731, Thermo Fisher Scientific), Neurotrace 640/660 nm (N21483, Thermo Fisher Scientific)
Validation	https://labchem-wako.fujifilm.com/europe/product/detail/W01W0101-1974.html<br>https://www.abcam.com/products/primary-antibodies/gfap-antibody-ab53554.html?productWallTab=ShowAll<br>https://www.thermofisher.com/order/catalog/product/N21483

Animals and other research organisms

Policy information about studies involving animals; ARRIVE guidelines recommended for reporting animal research, and Sex and Gender in Research

Laboratory animals	Rat: Long Evans, implanted at 5-7 months old<br>Mouse: C57BL/6, implanted at 2 months old
Wild animals	n/a
Reporting on sex	Rat: Female; Mouse: Male
Field-collected samples	n/a
Ethics oversight	All experimental and surgical procedures involving animals were approved by the local veterinary authorities of Canton Zurich, Switzerland, and were carried out in accordance with the guidelines published in the European Communities Council Directives 2010/63/EU.

Note that full information on the approval of the study protocol must also be provided in the manuscript.
